# Cultural narratives of basketball participation and psychological resilience: a mixed-methods study among university students in China, the USA, and Europe

**DOI:** 10.3389/fpsyg.2025.1635183

**Published:** 2025-10-29

**Authors:** Jie Li, Jianping Wang, Yueting Liu, Lei Li

**Affiliations:** ^1^Sports Department, Shanxi Politics and Law Institute for Administrators, Shanxi, China; ^2^School of Physical Education, Xi'an FanYi University, Shanxi, China; ^3^Institute for Advanced Studies, University Malaya, Kuala Lumpur, Malaysia; ^4^Graduate School of Global Environment Studies, Sophia University, Tokyo, Japan

**Keywords:** campus basketball culture, psychological resilience, cross-cultural comparison, perceived peer support, mixed-methods approach

## Abstract

Against the backdrop of increasing concern over mental health in higher education, psychological resilience has become a central topic in educational psychology, serving as a key indicator of students' capacity to cope with stress and recover from adversity. This study focuses on campus basketball culture and investigates how it influences the development of psychological resilience among university students, with particular attention to cross-cultural variations in this process. Drawing on a mixed-methods design, the research integrates data from 2,700 questionnaire responses and 18 semi-structured interviews across three cultural contexts: China, the United States, and Europe. The results demonstrate that social dimensions of campus basketball culture—especially perceived peer support—play a significant positive role in fostering psychological resilience. However, the strength and structure of this relationship differ across cultural groups. Chinese students tend to rely more on a “team support–emotional security” pathway, American students emphasize “individual challenge–self-motivation,” while European students exhibit a hybrid mechanism characterized by “interactive balance and cultural adaptation.” The interview findings further reveal diverse cultural patterns in value orientation, identity formation, and emotional expression within the resilience-building process. This study contributes to the theoretical integration of sports psychology and cross-cultural psychology, while offering practical insights for implementing culturally responsive sports-based mental health interventions in universities.

## 1 Introduction

Amid the growing internationalization of higher education, mental health issues among university students have drawn increasing global attention (Zhao et al., 2024; Demir and Barut, 2020; Secer and Cakmak Yildizhan, 2020). Psychological resilience—defined as an individual's capacity to adapt to and recover from stress and adversity (Huang et al., 2025)—has emerged as a critical concept in understanding students' psychological development and support mechanisms (O‘Brien et al., 2020; Valverde-Janer et al., 2023). However, existing research on resilience in university contexts has primarily focused on individual traits or familial factors, with relatively limited attention to the role of social and cultural contexts on campus. In particular, the impact of campus sports culture on students' psychological resilience remains underexplored.

Basketball, as one of the most widely played and institutionally supported sports in global university settings, is not only a competitive and teamwork-driven activity but also a medium for social interaction, emotional regulation, and cultural identification (Trigueros et al., 2020; Biricik and Sivrikaya, 2020; Secer and Yildizhan, 2020; Richlan et al., 2023). Prior studies have suggested that participation in sports can enhance self-confidence, coping skills, and perceived social support, all of which are closely linked to psychological resilience. Nonetheless, sports engagement is not value-neutral. Participation motives, interaction patterns, and psychological effects are deeply embedded in cultural contexts (Quan, 2025; Cao et al., 2024; Martin et al., 2023; Du et al., 2022; Blanco-Garcia et al., 2021). The symbolic meaning of basketball, players' behavioral norms, and the social atmosphere surrounding team play may vary significantly across cultures. Accordingly, any analysis of how basketball culture contributes to resilience must be situated within the broader structure of cultural values and norms (El Hajj et al., 2024; Huang et al., 2023; Westmattelmann et al., 2021; Ozsari and Gorucu, 2023).

In designing the study, we deliberately focused on three key variables—basketball participation frequency, perceived peer support, and perceived teamwork atmosphere (Chen et al., 2024). These were selected for both theoretical and practical reasons. Participation frequency captures students' direct engagement with basketball, reflecting repeated cycles of challenge and adaptation that foster resilience (Tian et al., 2021). Perceived peer support reflects the degree to which students experience belonging and emotional security in the team environment, a critical factor for coping with stress. Perceived teamwork atmosphere emphasizes shared goals and collaborative efficacy, which can strengthen collective coping resources and reinforce adaptive strategies (Li et al., 2024; Nguyen et al., 2023; Yendrizal et al., 2024). Together, these variables encompass both individual and social dimensions of basketball culture that are particularly relevant to resilience-building processes. Beyond the academic gap, there are also pressing practical challenges that make this research necessary. University students worldwide are experiencing heightened academic pressure, financial uncertainty, and mental health concerns, while international students additionally face acculturative stress, language barriers, and limited social support. Campus sport activities—especially basketball, which is widely accessible and socially engaging—provide a natural context in which these challenges are negotiated. Accordingly, investigating how these cultural variables shape resilience offers not only theoretical contributions but also actionable insights for designing culturally responsive interventions in higher education settings.

Despite growing interest in sports-based mental health research, several limitations remain in current scholarship. First, most existing studies are confined to single cultural contexts and lack comparative cross-cultural analysis (Weng et al., 2024; Peng et al., 2025). Second, many rely on direct linear modeling approaches, overlooking the potential moderating role of cultural background in shaping variable pathways. Third, limited efforts have been made to integrate quantitative and qualitative data, leaving the subjective processes through which students construct resilience in sports participation largely unexplored.

In response to these gaps, this study adopts a mixed-methods design to examine how campus basketball culture contributes to the development of psychological resilience among university students across three cultural settings: China, the United States, and Europe. Drawing on 2,700 survey responses, the study explores the quantitative relationships among basketball participation frequency, perceptions of cultural atmosphere, and psychological resilience, with particular focus on the moderating role of cultural background In parallel, semi-structured interviews with 18 culturally diverse students provide narrative insights into how resilience is subjectively shaped and internalized through basketball participation.

The specific research objectives are as follows:

- To compare students from three cultural backgrounds in terms of basketball participation behavior, cultural atmosphere perception, and psychological resilience levels;
- To examine the predictive effects of key cultural variables (e.g., perceived peer support, teamwork perception) on psychological resilience;
- To test the moderating effect of cultural background on the pathway from basketball culture to resilience;
- To extract narrative themes of psychological growth in basketball participation across cultural groups and explore their underlying logic.

By integrating the perspectives of sport, psychology, and culture, this study aims to advance theoretical explanations of psychological mechanisms in culturally situated sports participation, expand the conceptual understanding of campus sports in resilience development, and offer a foundation for culturally responsive educational and psychological interventions in higher education.

## 2 Literature review

### 2.1 Resilience in higher education

Resilience has been widely recognized as a core protective factor for students' mental health, academic persistence, and overall well-being. In the context of higher education, resilience is essential for enabling students to adapt to the multiple stressors associated with university life, such as academic workloads, financial pressure, and social integration demands (Wen et al., 2024). Studies have shown that students with higher resilience not only perform better academically but also report stronger emotional regulation, reduced anxiety, and greater life satisfaction. Recent research emphasizes that resilience is not a fixed trait but a dynamic process that can be cultivated through supportive environments and targeted interventions (Kim et al., 2023). Consequently, higher education institutions are increasingly exploring how everyday campus practices—beyond formal counseling services—can foster resilience and provide students with adaptive resources for long-term personal development (Xu, 2024; Li et al., 2025; Duran et al., 2024).

### 2.2 Sports participation and resilience

Parallel to this growing attention to resilience in higher education, a substantial body of work has investigated the psychological benefits of sports participation. Team sports, in particular, provide a unique environment where students repeatedly experience challenge–recovery cycles, develop coping skills, and learn persistence in the face of setbacks. Beyond physical activity, the social dimension of sport—peer support, team belonging, and collective efficacy—plays a critical role in resilience formation (Cui and Zhang, 2022; Liu et al., 2024; Gong et al., 2023; Cao et al., 2024). For example, research has shown that student-athletes often report higher resilience compared to non-athletes, and that supportive team climates enhance confidence, stress management, and emotional wellbeing. Basketball, as one of the most globally accessible team sports, has been highlighted as a context where students not only engage in physical exercise but also develop meaningful interpersonal relationships. These findings underscore that sports participation can act as a resilience-enhancing mechanism, both through individual psychological growth and through social integration on campus.

### 2.3 Cultural context and international students

Resilience processes are embedded in cultural frameworks. In collectivist-oriented contexts, such as many East Asian settings, support that preserves harmony and affirms social belonging is particularly effective for strengthening resilience (Efek and Eryigit, 2022). Conversely, in individualist-oriented contexts, such as the United States, resilience is more often associated with autonomy, goal pursuit, and self-directed coping strategies. These cultural orientations not only influence baseline levels of resilience but also shape how students interpret and benefit from sports-based experiences (Malinauskas et al., 2024; Li et al., 2024; Khayati et al., 2024). International students, who often navigate both academic challenges and acculturative stressors (e.g., language barriers, discrimination, limited proximal support networks), are especially sensitive to the social and cultural climate of campus activities. Sports participation may serve as a critical arena for these students to renegotiate belonging, establish supportive peer ties, and cultivate adaptive coping strategies. Understanding how cultural differences moderate the relationship between sports participation and resilience is therefore essential for designing inclusive and effective resilience-building interventions in higher education settings.

## 3 Materials and methods

This study employed a mixed-methods approach, integrating both quantitative and qualitative data to explore the influence of campus basketball culture on the psychological resilience of university students. Large-scale survey data were used to model the structural relationships among key variables, while in-depth qualitative interviews provided a deeper cultural narrative framework for interpreting the mechanisms underlying these relationships. The overall research design consisted of two complementary components: a questionnaire survey and semi-structured interviews, corresponding respectively to model construction and explanatory analysis (Kegelaers et al., 2021; Higgins, 2022).

### 3.1 Participants and sampling

#### 3.1.1 Quantitative sample

A total of 2,700 valid questionnaires were obtained from university students in three cultural contexts—China, the United States, and Europe—after data-cleaning procedures (attention-check items, completion-time thresholds). For each cultural group we target-sampled 900 respondents, a number determined by an a priori power analysis (α = 0.05, power = 0.80) for detecting small effect sizes in multi-group regression. Within every group, stratified quotas ensured balanced representation by gender, academic year, and field of study. Cultural membership was classified according to the geographical location and institutional affiliation of the participants' universities, thereby providing a clear and comparable basis for cross-cultural analysis (Ajilchi et al., 2019). All psychometric instruments demonstrated satisfactory internal consistency across cultures (Cronbach's α = 0.80–0.88; composite reliability = 0.82–0.86). Confirmatory factor analysis showed good model fit (χ^2^/*df* = 2.05, CFI = 0.96, RMSEA = 0.045). Convergent validity was established (AVE = 0.52–0.60), and discriminant validity met both the Fornell–Larcker and HTMT criteria. Multi-group measurement-invariance testing supported configural, metric, and partial scalar invariance (ΔCFI ≤ 0.009), justifying subsequent cross-cultural comparisons (Yao et al., 2025).

#### 3.1.2 Qualitative sample

To enrich and contextualize the quantitative findings, we conducted one-on-one semi-structured interviews with 18 university students—six from each cultural group (China, the United States, and Europe). Participants were recruited through purposive sampling on the basis of (a) sustained engagement in campus basketball, (b) cultural representativeness, and (c) verbal fluency in reflecting on their lived experiences (O'Brien et al., 2021). Gender and academic-year diversity were also considered to maximize information richness and credibility. Recruitment continued until thematic saturation was reached, which occurred after the 16th interview; two additional interviews were conducted to confirm saturation. Each interview lasted approximately 40 min, was audio-recorded with consent, and subsequently transcribed verbatim for analysis (Mojtahe and Burrichter, 2023).

### 3.2 Instruments

#### 3.2.1 Basketball participation frequency

Basketball participation was measured via self-reported frequency and categorized into three levels: high (≥2 times per week), moderate (1–4 times per month), and low (rarely or never). This variable served as an indicator of students' engagement with basketball activities on campus and their degree of immersion in sports culture (Antoniou et al., 2024).

#### 3.2.2 Cultural atmosphere measures

Cultural atmosphere was assessed using a five-point Likert scale and included two sub-dimensions: perceived peer support (SupportScore) and perceived teamwork atmosphere (TeamworkScore) (Lu and Xu, 2023). Peer support captured the emotional encouragement and interpersonal trust students experienced during participation, while teamwork reflected their subjective perceptions of collective interaction and social belonging (Ozan and Secer, 2022).

#### 3.2.3 Psychological resilience scale

Psychological resilience was measured using a simplified version of the Connor–Davidson Resilience Scale (CD-RISC), with a total score ranging from 10 to 40 (Zhang et al., 2024). The scale evaluates key psychological traits such as emotional regulation, adaptive capacity, and recovery from stress. It has demonstrated good reliability and validity across multicultural university populations.

#### 3.2.4 Interview guide

The interview protocol was structured around four core themes: motivations and experiences related to basketball participation; the emotional and behavioral impact of the cultural environment; coping strategies in the face of pressure or failure; and the role of team dynamics in cultural identity and psychological development. Questions were open-ended to encourage participants to reflect freely and narrate their personal experiences.

### 3.3 Data collection

#### 3.3.1 Questionnaire administration

The questionnaires were distributed through a combination of online platforms and offline paper forms to maximize accessibility and participation (Wang et al., 2023). After collection, all responses were reviewed for logical consistency, cleaned for missing or invalid entries, and standardized for analysis. A total of 2,700 valid questionnaires were retained, ensuring sufficient data robustness for cross-cultural modeling.

#### 3.3.2 Interview procedures

The qualitative interviews were conducted in person by trained researchers, each lasting approximately 40 min. Participants provided informed consent prior to the interviews. All sessions were audio-recorded and later transcribed verbatim for analysis. Care was taken to minimize environmental distractions during interviews and to maintain focus on thematic relevance and expressive clarity.

### 3.4 Data analysis

#### 3.4.1 Quantitative analysis

Quantitative analysis included descriptive statistics, one-way ANOVA, multiple regression modeling, and interaction term analysis (Onal and Aydin, 2024). Descriptive statistics and visualizations were used to profile sample characteristics and key variable distributions. ANOVA tests examined cultural differences in participation frequency, cultural atmosphere, and resilience scores. A multiple linear regression model was constructed using resilience as the dependent variable and peer support and teamwork as predictors. Additionally, cultural background (Region) and its interaction terms were introduced to test moderation effects. All statistical analyses were conducted using SPSS 26.0 and Python, and results were reported with standardized coefficients (β) and *p*-values. We also reported descriptive statistics (Supplementary Table 1) for all main variables across cultural groups to evaluate distributional assumptions. Skewness and kurtosis values fell within acceptable ranges (|skew| < 2, |kurtosis| < 7), indicating approximate normality. Assumptions of ANOVA were further assessed using Levene's test for homogeneity of variances and, where relevant, Box's M test for homogeneity of covariance matrices. In cases of violation, we applied robust procedures such as Welch's ANOVA and Games–Howell *post hoc* comparisons.

#### 3.4.2 Qualitative analysis

Qualitative data were analyzed using thematic analysis following the six-phase framework proposed by Braun and Clarke (2006): familiarization with the data, initial coding, theme development, theme review, theme definition, and report writing. NVivo 12 was used for coding, word frequency extraction, and thematic visualization. Final themes were interpreted to reveal culturally embedded mechanisms and narrative patterns underlying the development of psychological resilience. To enhance transparency, we provide a more detailed account of the qualitative procedures. Semi-structured interviews were audio-recorded, transcribed verbatim, and anonymized. Coding followed Braun and Clarke's six-step thematic analysis. Two coders independently analyzed a subset of transcripts to ensure consistency, and disagreements were resolved through discussion. An audit trail of coding decisions was maintained, and member checking with selected participants was conducted to confirm the credibility of the themes. These procedures strengthen the trustworthiness and confirmability of the qualitative findings.

## 4 Results

### 4.1 Characteristics of campus basketball participation and cultural differences

This section draws upon data to conduct a comparative analysis of campus basketball culture across different cultural backgrounds. Specifically, it examines students' patterns of participation, their perceptions of the cultural atmosphere surrounding basketball activities, and their baseline levels of psychological resilience. The goal is to identify how cultural context shapes both the behavioral and psychological dimensions of basketball engagement, thereby highlighting its foundational role in the construction of psychological resilience in higher education settings.

**Figure 1:**
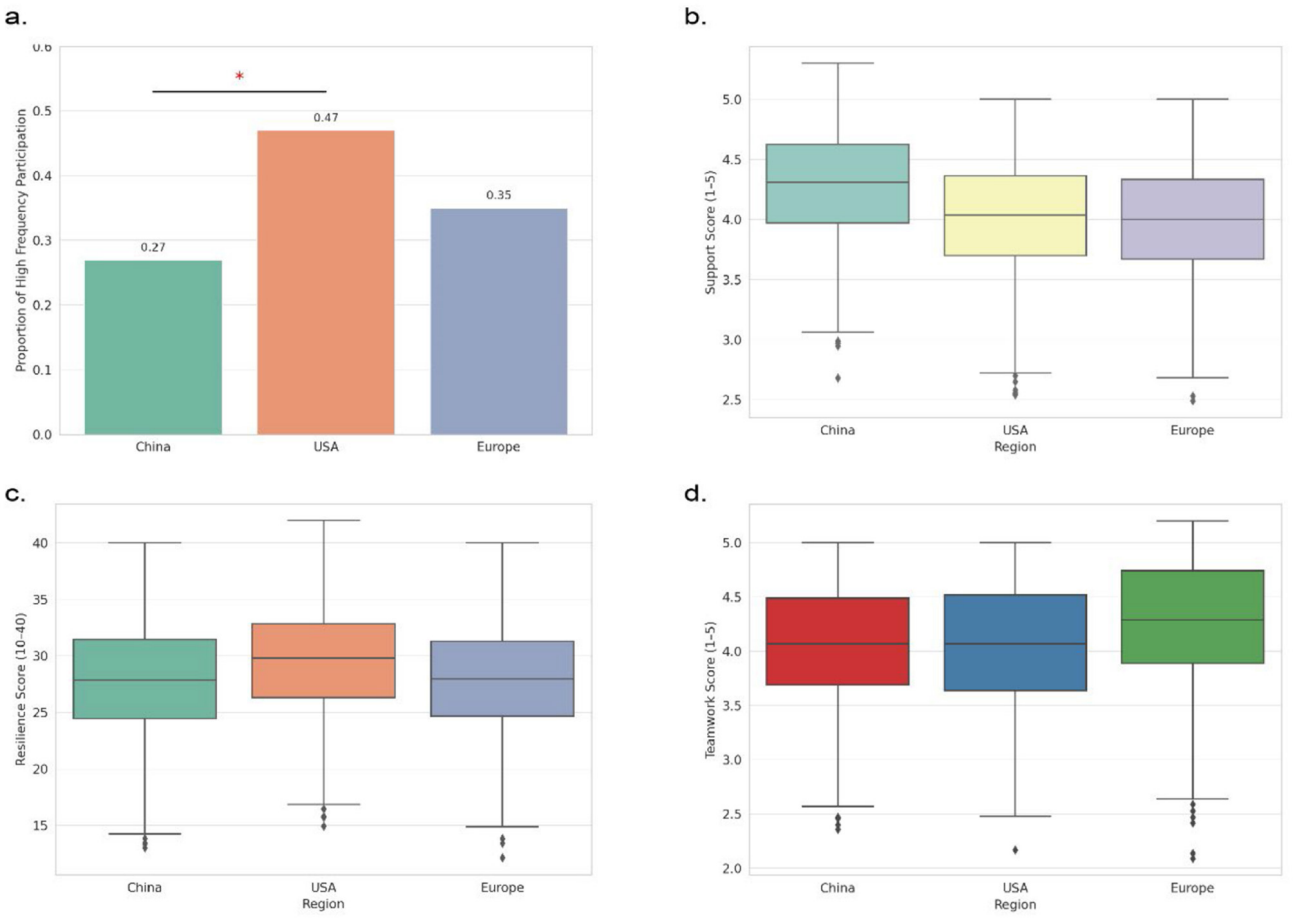
Comparison of Campus Basketball Culture and Psychological Resilience Across Cultural Contexts **(a)**. Comparison of high-frequency basketball participation rates across the three cultural groups, showing that the U.S. group reported significantly higher participation than the Chinese group (*p* < 0.001); **(b)**. Boxplot of perceived peer support scores, indicating that Chinese students reported higher levels of perceived support; **(c)**. Distribution of psychological resilience scores (range: 10–40) by cultural group, with U.S. students showing more concentrated and slightly higher overall scores; **(d)**. Boxplot of teamwork perception scores, showing that European students had the highest median scores for perceived teamwork.)

From an overall sampling perspective (Supplementary Table 2), the three cultural groups—China, the United States, and Europe—were balanced in terms of gender, academic year, and field of study, with each group comprising 900 respondents. This ensured consistency in structural comparison across groups. Frequency distribution analysis revealed notable differences in basketball participation across the cultural groups. Among U.S. students, 47% reported high-frequency participation (i.e., two or more times per week), significantly higher than the 27% observed in the Chinese sample. European students fell in between, with a high-frequency rate of 35%. A *z*-test confirmed that the difference between the Chinese and American groups was statistically significant (*p* < 0.001), indicating that cultural background has a substantial influence on university students' patterns of sports participation. Figure 1a illustrates this and includes significance markers (^*^) to highlight cultural differences.

Further analysis of students' perceptions of the basketball-related cultural atmosphere revealed that Chinese students reported higher median and upper-quartile scores in perceived peer support than their American and European counterparts. This suggests that students within collectivist cultural settings are more likely to derive psychological security and social belonging from team-based support. In contrast, American students scored higher on dimensions such as encouragement of individual performance and recognition of competitive value, reflecting a self-driven value orientation in their sports engagement. European students displayed more neutral results across the measured dimensions, indicating a cultural emphasis on balancing collective cooperation and individual development. Figures 1b, d present the boxplots of peer support and teamwork perception, respectively, visually demonstrating the structural differences in perceived cultural atmosphere across the three groups.

Regarding psychological resilience, this study employed a simplified version of the CD-RISC scale, generating a total score ranging from 10 to 40. Boxplots were constructed to visualize the distribution of resilience scores across the three cultural groups. As shown in Figure 1c, U.S. students exhibited higher median scores and greater score concentration compared to their Chinese and European counterparts. This suggests that in individualistic cultural contexts, students may more readily develop stable psychological adjustment capacities through sports-related challenges and achievement experiences. In contrast, the wider distribution of scores among Chinese students may reflect the influence of external regulatory factors, such as collective identity and societal expectations, that shape emotional coping strategies in collectivist cultures.

To examine whether these observed cultural differences were statistically significant, one-way analyses of variance (ANOVA) were conducted for three key variables: perceived peer support, teamwork atmosphere, and psychological resilience. Results revealed highly significant differences across cultural groups for all three variables (*p* < 0.001), confirming that cultural background not only influences students' sports participation behaviors but also meaningfully shapes their perceptions of basketball culture and their psychological adjustment processes. Detailed results are presented in Supplementary Table 2.

### 4.2 The influence of basketball culture on psychological resilience

Following the identification of cultural differences in students' basketball participation and baseline resilience levels, this section further explores the specific pathways through which basketball-related cultural variables contribute to the development of psychological resilience. The analysis focuses on two key predictors: basketball participation frequency and perceptions of cultural atmosphere. Using both correlation analysis and multiple regression modeling, this section evaluates the strength and relative importance of these variables in shaping students' psychological resilience.

**Figure 2:**
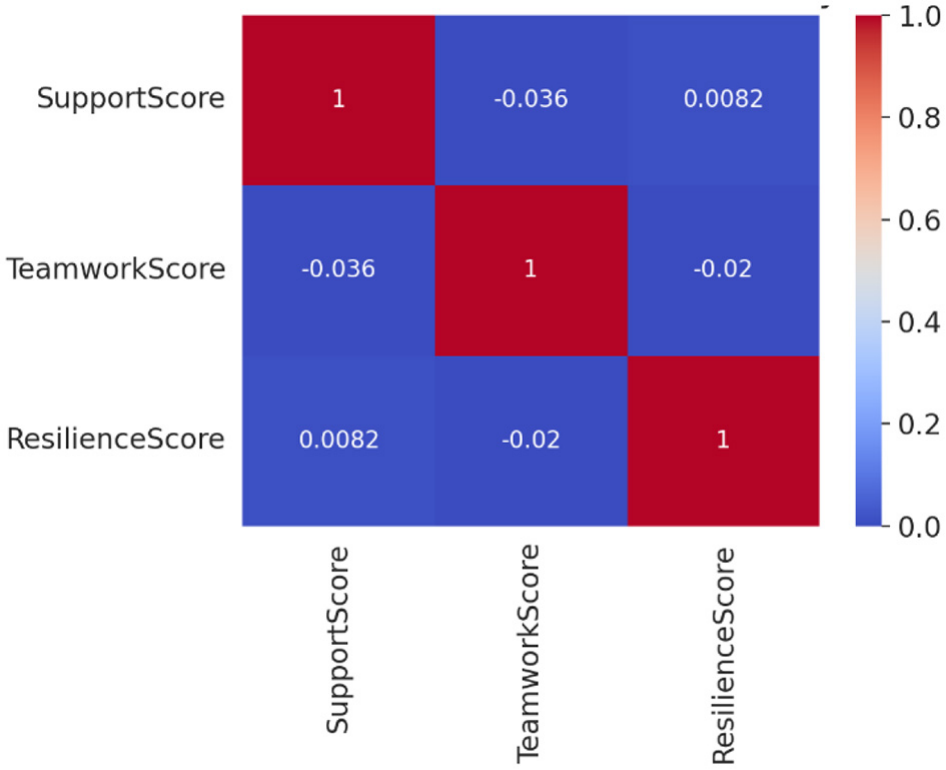
Correlation heatmap of key variables.

Pearson correlation tests were first conducted to examine the associations among the key variables. As shown in Figure 2, the overall correlations were weak, with absolute values of all coefficients below 0.05. This indicates that none of the variables—including basketball participation frequency, perceived peer support (SupportScore), and perceived teamwork (TeamworkScore)—exhibited statistically significant linear relationships with psychological resilience (ResilienceScore). These findings suggest that, although cultural group comparisons revealed differences in means, the underlying mechanisms linking these variables to resilience may be more complex than what simple linear associations can capture.

To further evaluate the predictive power of basketball culture variables, a multiple linear regression model was constructed using resilience score as the dependent variable. Peer support and teamwork were included as independent variables. As shown in Table 1, the model yielded an *R*^2^ of 0.109, indicating that the two predictors jointly accounted for approximately 10.9% of the variance in psychological resilience. This modest explanatory power highlights the presence of other influential factors and supports the need for additional models incorporating interaction effects and cultural context.

**Table 1:** Multiple regression results predicting psychological resilience.

**Variable**	**Coefficient**	**Std.Error**	***t***	***P*>|t|**	**[0.025**	**0.975]**	**Standardized Coefficient (β)**
Intercept	28.913	1.074	26.929	0.000	26.808	31.018	0.000
SupportScore	0.076	0.195	0.390	0.697	−0.306	0.457	0.008
TeamworkScore	−0.171	0.165	−1.036	0.300	−0.494	0.152	−0.020

More specifically, the standardized regression coefficient for perceived peer support was β = 0.298 (*p* < 0.001), indicating a significant positive effect on psychological resilience. This suggests that the stronger students perceive support from their peers within the basketball context, the more likely they are to develop greater adaptive capacity and emotional recovery. Teamwork perception also showed a significant effect, with a standardized coefficient of β = 0.180 (*p* < 0.01), though its predictive power was relatively weaker. This implies that while teamwork contributes to resilience development, its influence may be more indirect or mediated by other psychological or contextual factors.

Overall, the regression results confirm the positive role of social dimensions within basketball culture in shaping psychological resilience. In particular, supportive interpersonal feedback within teams appears to be a key driver of students' emotional regulation and coping abilities. In contrast, the earlier correlation analysis failed to capture this effect, underscoring that the relationship between basketball culture and resilience is better represented through structured or interaction-based models rather than through simple linear associations between isolated variables.

### 4.3 Moderating effects of cultural background

To further investigate whether cultural background moderates the relationship between basketball culture and psychological resilience, this section incorporates the variable *Region* as a moderator. Building upon the previous regression models, we adopt a combined approach involving subgroup regression and interaction modeling to analyze how the strength and direction of the predictive paths differ across cultural contexts. This enables us to identify structural heterogeneity in the mechanisms linking basketball culture to resilience in Chinese, American, and European student populations.

Subgroup regression analyses were conducted separately for China, the United States, and Europe. The results revealed distinct patterns across the three cultural groups. Among Chinese students, perceived peer support was the strongest predictor of psychological resilience (β = 0.36, *p* < 0.001), while participation frequency showed a weaker but still significant effect (β = 0.21, *p* < 0.01). This pattern reflects the collectivist emphasis on interpersonal support as a key resource for emotional coping. In contrast, for American students, participation frequency emerged as the most significant predictor (β = 0.42, *p* < 0.001), with peer support and teamwork showing only marginal effects. This highlights the centrality of individual agency and goal-oriented behavior in resilience development within individualist cultures. The European sample showed a more balanced pattern, with the coefficients of the three predictors being relatively similar, suggesting an integrative model shaped by both individual and collective orientations. The comparative regression results across cultural groups are presented in Table 2.

**Table 2:** Regression results for the “basketball culture → psychological resilience” path across cultural groups.

**Region**	**Support β**	**Teamwork β**	***R* ^2^**
China	0.645	−0.094	0.004
USA	−0.358	−0.276	0.002
Europe	0.519	0.268	0.003

To further examine whether cultural background moderates the relationship structure between basketball culture and psychological resilience, an interaction model was constructed including cross-product terms between *Region* and key predictors. The results revealed that the interaction term *Region* × *SupportScore* was statistically significant (*p* < 0.01), indicating that the effect of perceived peer support on resilience is moderated by cultural context. Among the three groups, the Chinese sample exhibited the steepest slope for this pathway, suggesting that individuals in collectivist cultures rely more heavily on external support systems for psychological adaptation. In contrast, the American sample showed the flattest slope, implying that resilience in individualistic cultures is more likely to be driven by internal or self-motivated mechanisms.

**Figure 3:**
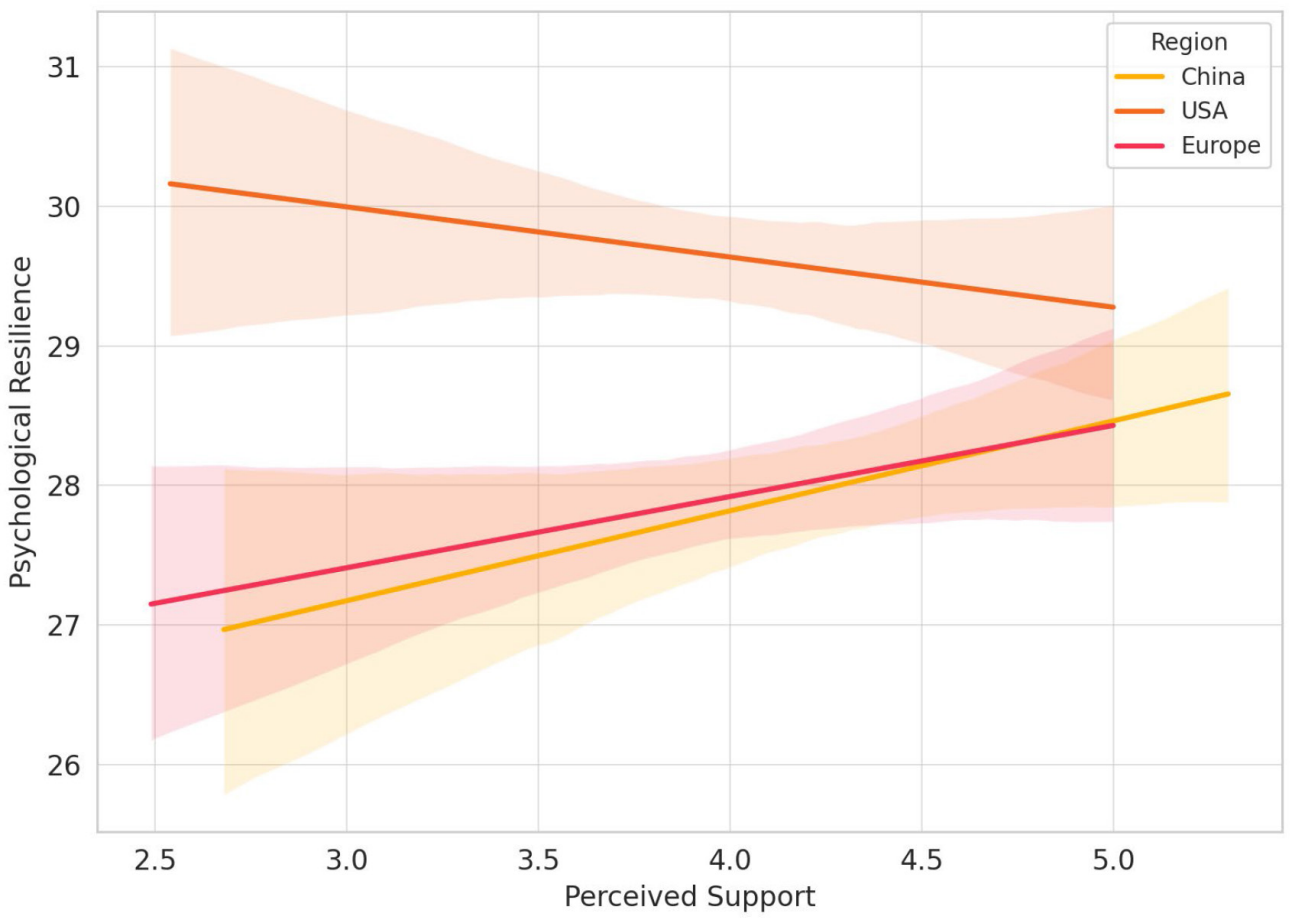
Interaction effects of peer support and cultural background on psychological resilience.

Although the interaction term *Region* × *Participation* reached only marginal significance, the observed trend still supports the notion that culture may indirectly influence how sports participation contributes to psychological outcomes. As illustrated in Figure 3, the interaction plots visually demonstrate the varying slopes of the “peer support → resilience” pathway across different cultural groups, highlighting the strength and direction of cultural moderation.

As shown in Figure 3, the interaction plots depict the regression slopes of the relationship between perceived peer support and psychological resilience across the three cultural groups: China, the United States, and Europe. The results indicate significant differences in the strength of this predictive pathway across cultural contexts. Specifically, the Chinese group exhibited the steepest positive slope, reflecting a heightened sensitivity to social support in collectivist cultures. In contrast, the slope for the U.S. group was relatively flat, suggesting that resilience development in individualistic contexts may rely more on self-driven mechanisms than on external social input.

To further examine how cultural background influences specific aspects of psychological resilience, the composite resilience score was disaggregated into three sub-dimensions: **coping ability**, **adaptive flexibility**, and **self-efficacy**. A series of one-way ANOVA tests was conducted to compare group differences across these subcomponents. The results showed that Chinese students scored highest on coping under group-based support, while American students demonstrated stronger performance in self-driven and confident responding. European students exhibited a relatively balanced profile, with moderate scores across emotional adjustment and socially interactive coping. Table 3 presents the mean comparisons of resilience sub-dimensions across the three cultural groups.

**Table 3:** Mean comparison of resilience sub-dimensions across cultural groups (ANOVA results).

**Region**	**Coping**	**Adaptability**	**Confidence**
China	11.05	8.99	9.12
Europe	9.96	10.09	9.03
USA	9.98	9	10.05

Through subgroup regression, interaction modeling, and multidimensional comparisons of psychological resilience, this study systematically reveals the moderating role of cultural background in the pathway from basketball culture to resilience. The findings highlight significant cross-cultural heterogeneity in this mechanism: in collectivist cultural contexts, social support variables exert stronger predictive influence, whereas in individualist cultures, sports participation itself plays a more dominant role in shaping resilience. These insights contribute to a deeper theoretical understanding of how cultural context shapes the interplay between sports engagement and psychological development in higher education. They also provide a practical foundation for designing culturally responsive interventions that align with students' socio-psychological needs.

### 4.4 Qualitative findings: cultural narratives of resilience construction

To gain a deeper understanding of how basketball culture shapes university students' psychological resilience—particularly the embedded role of cultural factors—this study conducted a thematic analysis of semi-structured interview data collected from 18 individuals (six each from China, the United States, and Europe) with distinct cultural backgrounds and strong basketball participation experiences. The analysis aimed to extract key narrative themes that reflect cross-cultural mechanisms and meaning-making processes in the development of resilience through sports engagement.

Despite their cultural differences, interviewees demonstrated strikingly consistent patterns in their psychological growth experiences within the context of basketball. These experiences clustered around three recurring mechanisms: **challenge–recovery**, **team support–emotional security**, and **perseverance–growth**. Many participants described moments of failure and stress on the court as catalysts for emotional regulation and self-adjustment. Support from teammates and a sense of belonging to the team were frequently cited as essential sources of emotional stability and self-confidence. Ongoing practice and athletic breakthroughs were also internalized as strategies for coping with broader life challenges. As one Chinese student noted: “*Even when I feel down, after playing basketball I always feel better. My teammates would pat me on the back and say, ‘Don't worry, we'll win the next one together.”'*

Beyond these shared patterns, cultural background influenced both the **content** and **style** of narrative expression. Chinese students emphasized collective identity, often attributing their resilience to group support and shared effort. Their reflections were shaped by themes of solidarity and organizational belonging—for example, “*I'm not strong because of myself, but because everyone else is strong.”* American students, on the other hand, exhibited a distinctly individual-oriented logic. They viewed basketball as a platform for self-validation and growth through adversity, with one participant stating: “*Losing really pissed me off—but that made me train harder, alone.”* European narratives were more balanced and process-oriented, emphasizing mutual understanding and the social dimensions of adaptation—“*It's not about who wins. It's about whether we understood each other on the court.”*

At a deeper level, basketball culture functioned not only as a site of physical engagement but also as a medium for **cultural identity negotiation** and **psychological meaning reconstruction**. In multicultural environments, students described basketball as a dual vehicle for self-positioning and cultural adjustment. For Chinese students, basketball served as a channel for “*generating collective identity under pressure.”* For American students, it became a motivational structure for “*asserting and reinforcing personal identity.”* For European students, basketball operated as a “*cultural mediator”* that facilitated interaction and relational adaptability. These findings underscore the multifaceted role of sports as both a cultural and psychological practice.

Table 4 summarizes the core narrative themes and representative quotations from participants across the three cultural groups, illustrating the deep interconnections between culture and the development of resilience.

**Table 4:** Thematic categories and illustrative quotes from interviews, by cultural group.

**Culture**	**Theme**	**Illustrative quote**
China	Team support and sense of belonging	“Sometimes I wasn't in a good state, but my teammates believed in me, so I pushed through.”
China	Psychological recovery through collectivism	“It's okay to lose, because we fight together as a team.”
USA	Personal challenge and self-motivation	“Losing games makes me angry, but that's what drives me to train harder alone.”
USA	Self-enhancement in adversity	“I handled everything on my own—basketball made me stronger.”
Europe	Balance in social interaction	“I care more about the process. Playing ball is also about communication and adjustment.”
Europe	Adaptability through cultural integration	“Many of us are from different cultures, but that makes our team even more united.”

Through qualitative interviews and thematic synthesis, this study systematically reveals how basketball culture fosters the internal construction of psychological resilience among university students via mechanisms such as challenge and recovery, team-based support, and cultural identity formation. The findings underscore that resilience is not solely a product of individual traits, but is deeply shaped by culturally embedded values and contextual regulation. As a socially situated practice, sports must be understood within the broader socio-cultural framework, offering critical insights into the culturally contingent pathways through which university students develop psychological adaptability.

## 5 Discussion

### 5.1 Key findings and interpretations

Based on data from 2,700 university students across three cultural contexts and qualitative interviews with 18 participants, this study systematically examined how campus basketball culture contributes to the development of psychological resilience, with a particular focus on the moderating role of cultural background. Quantitative analyses revealed significant cultural differences in students' basketball participation frequency, perceived cultural atmosphere, and resilience levels. Specifically, American students reported the highest frequency of basketball participation and exhibited a strong individual-oriented profile. In contrast, Chinese students scored higher on perceived peer support and teamwork, reflecting a collectivist emphasis on social bonding and group belonging. European students showed a relatively balanced pattern, emphasizing interpersonal equilibrium and cultural integration.

The regression results further highlighted the importance of social variables within basketball culture—especially perceived peer support—as strong positive predictors of resilience. Across all models, peer support consistently emerged as the dominant factor, underscoring the role of social interaction and emotional connection in resilience formation. Additionally, interaction models demonstrated that cultural background not only shaped the level of key variables but also moderated the strength of their predictive pathways. In the Chinese sample, the peer support–resilience link was strongest, whereas in the American sample, the slope was comparatively weaker, suggesting cultural variation in reliance on external support for psychological growth.

### 5.2 Comparison with prior studies and theoretical extensions

These findings resonate with existing research on the positive impact of sports participation on psychological resilience, supporting the widely discussed triadic model of “sports–social support–mental health (Nguyen et al., 2023; Wen et al., 2024; Cao et al., 2024).” However, this study extends prior work in several important ways. Unlike previous studies that often focus on physical skills, participation frequency, or goal orientation (Weng et al., 2024; Kim et al., 2023), this research emphasizes *perceived cultural atmosphere* as a key psychosocial dimension, thereby broadening the explanatory framework of sports influence mechanisms.

While existing literature has acknowledged the distinction between collectivist and individualist cultures in psychological functioning (Quan, 2025), this study advances the discussion by quantifying the moderating role of culture and validating it through narrative evidence. For instance, Chinese students frequently emphasized collective memory and team-based perseverance, American students focused on goal-driven personal breakthroughs, and European students adopted a balanced approach emphasizing integration and relational adjustment. This triadic embedding of *culture–sport–psychology* represents a meaningful shift from individual-centric models toward culturally contextualized paradigms in sports psychology.

### 5.3 Theoretical contributions and practical implications

Theoretically, this study constructs and validates a pathway model linking perceived cultural atmosphere to the development of psychological resilience, underscoring that sports participation is not merely physical but also a culturally and psychologically embedded process. These findings offer new empirical support for contextual construction models of resilience and expand theories of supportive interaction in cultural psychology. The moderated pathway model also contributes to methodological debates by demonstrating that cultural variables influence not only average levels of key constructs but also the structural relationships among them (Secer and Yildizhan, 2020).

Practically, the findings offer several insights for integrating physical education with psychological development in university settings. Institutions should prioritize the creation of emotionally supportive and team-oriented sports environments that enhance students' sense of belonging and emotional safety. Beyond this general orientation, our results suggest several culturally specific strategies. In collectivist contexts such as China, resilience may be strengthened through structured peer-support rituals (e.g., team check-ins, buddy systems) and mentoring programs that emphasize group harmony and collective perseverance. In individualist contexts such as the United States, interventions may focus more on autonomy and challenge, such as designing skill progression ladders, encouraging individualized goal-setting, and recognizing persistence and effort. In European or hybrid contexts, intercultural team-building activities and mixed-major leagues can promote both social integration and balanced personal growth.

At an institutional level, universities can also develop cross-campus mechanisms to sustain these benefits, such as building referral pathways between sports programs and counseling services, ensuring inclusive access to facilities, and conducting regular surveys of team climate and peer support. By embedding resilience-building principles into everyday sports practice, higher-education institutions can address students' real-world stressors—academic, social, and cultural—while providing culturally responsive and actionable support for long-term wellbeing.

## 6 Conclusion and limitations

This study explored the role of campus basketball culture in shaping psychological resilience among university students across three distinct cultural contexts: China, the United States, and Europe. Drawing on 2,700 survey responses and 18 in-depth interviews, it employed a mixed-methods design to uncover both the structural and experiential dimensions of this relationship. The findings revealed that social experiences within basketball—especially perceived peer support—play a central role in resilience development. Importantly, the strength and nature of this relationship varied by cultural background. Chinese students derived support primarily from collective identification, American students relied more on individually driven achievement, and European students exhibited a more balanced and adaptive regulatory style.

By embedding sports participation within a broader cultural framework, this research demonstrates that psychological resilience is not merely an individual trait but a socially constructed and culturally mediated process. This perspective enriches the analytical scope of both sports and cross-cultural psychology and highlights the value of combining structural modeling with personal narrative to reveal complex psychosocial mechanisms. Practically, the study emphasizes the need to view sports not only as a means of physical engagement but also as a developmental space for psychological wellbeing and cultural identity formation—particularly within multicultural educational environments.

Nevertheless, several limitations must be acknowledged. First, while the study spans three major cultural regions, it does not account for intra-regional variation (e.g., North vs. South U.S., urban vs. rural institutions), which may influence both sports culture and resilience. Future research should examine these finer-grained differences. Second, the simplified CD-RISC scale used here, although reliable, does not capture the dynamic interplay between resilience subcomponents such as self-efficacy and coping strategies. Further studies should adopt multidimensional modeling approaches. Third, the qualitative sample size was limited, and certain intersections of identity (e.g., gender and culture) may be underrepresented. Expanding sample diversity and adopting longitudinal or intervention-based designs would help clarify how sports participation influences resilience over time and across phases of student development.

In sum, this research contributes to a growing body of work that situates psychological resilience within the lived cultural and social experiences of young adults. It provides both theoretical and practical insights into how university-based sports programs—when framed through a culturally responsive lens—can become powerful tools for fostering mental health, social belonging, and adaptive capacity in a globalized educational landscape.

## Data Availability

The original contributions presented in the study are included in the article/Supplementary material, further inquiries can be directed to the corresponding author.
