# Supplementary material for: Cultural narratives of basketball participation and psychological resilience: a mixed-methods study among university students in China, the USA, and Europe
Source: Front Psychol. 2025 Oct 29;16:1635183. doi: 10.3389/fpsyg.2025.1635183 (PMC12605183; doi:10.3389/fpsyg.2025.1635183)
Supplement: Supplementary file 1 [file Table_1.docx]

Table S1. Descriptive Statistics

| Region | Variable | mean | std | min | max | skewness | kurtosis |
| --- | --- | --- | --- | --- | --- | --- | --- |
| China | SupportScore | 4.30 | 0.48 | 2.68 | 5.30 | -0.02 | -0.30 |
| China | ResilienceScore | 28.01 | 5.17 | 13.04 | 40.00 | -0.06 | -0.28 |
| China | TeamworkScore | 4.06 | 0.57 | 2.36 | 5.00 | -0.24 | -0.38 |
| Europe | SupportScore | 4.00 | 0.48 | 2.49 | 5.00 | -0.14 | -0.26 |
| Europe | ResilienceScore | 27.92 | 4.88 | 12.12 | 40.00 | -0.05 | 0.04 |
| Europe | TeamworkScore | 4.29 | 0.58 | 2.09 | 5.20 | -0.32 | -0.15 |
| USA | SupportScore | 4.03 | 0.49 | 2.54 | 5.00 | -0.16 | -0.18 |
| USA | ResilienceScore | 29.62 | 4.91 | 14.96 | 42.00 | -0.13 | -0.17 |
| USA | TeamworkScore | 4.06 | 0.59 | 2.17 | 5.00 | -0.26 | -0.54 |

Table S2. Frequency Distribution of Basketball Participation Across Three Cultural Groups

| **Region** | **High** | **Low** | **Medium** | **All** |
| --- | --- | --- | --- | --- |
| **China** | 255 | 183 | 462 | 900 |
| **Europe** | 309 | 173 | 418 | 900 |
| **USA** | 411 | 121 | 368 | 900 |
| **All** | 975 | 477 | 1248 | 2700 |

Table S3. One-Way ANOVA Results for Key Variables Across Cultural Groups

| **Variable** | **F-statistic** | **p-value** |
| --- | --- | --- |
| **SupportScore** | 108.611 | 0 |
| **ResilienceScore** | 33.247 | 0 |
| **TeamworkScore** | 46.907 | 0 |

**Note:** This table summarizes the results of one-way analysis of variance (ANOVA) tests comparing three cultural groups (China, USA, Europe) on peer support (SupportScore), teamwork perception (TeamworkScore), and psychological resilience (ResilienceScore). All three variables showed statistically significant differences across groups (*p* < 0.001), indicating the presence of strong cross-cultural effects on both cultural atmosphere and resilience-related outcomes.
